# Translation and adaptation of the EORTC QLQ-LC 29 for use in Chinese patients with lung cancer

**DOI:** 10.1186/s41687-021-00397-9

**Published:** 2021-11-10

**Authors:** Wei Dai, Yaqin Wang, Yangjun Liu, Xing Wei, Ahmed M. Y. Osman, Cecilia Pompili, Michael Koller, Qifeng Wang, Yi Wang, Jun Ge, Tianpeng Xie, Qiang Li

**Affiliations:** 1 Department of Thoracic Surgery, Sichuan Cancer Hospital & Institute, Sichuan Cancer Center, School of Medicine, University of Electronic Science and Technology of China, Chengdu, China; 2 Department of Molecular Medicine and Surgery, Karolinska Institutet, Stockholm, Sweden; 3 School of Pharmaceutical Science and Technology, Tianjin University, Tianjin, China; 4 Center for Social Science Survey and Data, Tianjin University, Tianjin, China; 5 Section of Patient Centred Outcomes Research, Leeds Institute for Medical Research at St James’s, University of Leeds, Leeds, UK; 6 Department of Thoracic Surgery, Leeds Teaching Hospitals NHS Trust, Leeds, UK; 7 Center for Clinical Studies, University Hospital Regensburg, Regensburg, Germany; 8 Department of Radiation Oncology, Sichuan Cancer Hospital & Institution, Sichuan Cancer Center, School of Medicine, University of Electronic Science and Technology of China, Chengdu, China; 9 Department of Medical Oncology, Sichuan Cancer Hospital & Institution, Sichuan Cancer Center, School of Medicine, University of Electronic Science and Technology of China, Chengdu, China

**Keywords:** Lung cancer, Quality of life, LC29, Translation, Cultural adaptation, Simplified Chinese

## Abstract

**Background:**

The latest European Organisation for Research and Treatment of Cancer (EORTC) Quality of Life Questionnaire-Lung Cancer 29 (QLQ-LC29) has been translated and validated in several languages but not yet in simplified Chinese. This study aimed to translate this questionnaire into simplified Chinese and adapt it for use in Chinese patients with lung cancer.

**Methods:**

The translation and adaptation process followed the EORTC translation procedure, and consisted of eight steps, namely, translation preparation, forward translations, reconciled translation, back translations, a back translation report, proofreading, pilot testing, and finalisation. The pilot testing included 10 patients with lung cancer.

**Results:**

We obtained the permission to perform the EORTC QLQ-LC29 translation work on November 17, 2020. Thereafter, it took 3 weeks to complete the forward translations, reconciled translation, and back translations. After several rounds of discussion with the EORTC Translation Unit, 19 items used the existing translations from the EORTC Item Library (a database of EORTC questionnaire items and their translations), and 10 items were translated from scratch. The 10 patients included in the pilot testing phase had a median age of 64 years (range 31–69 years); five were male, five had an educational level of high school or above, and six had undergone surgery. Eight items received comments from patients (six items by one patient alone and the other two items by three patients). No patients commented on the instructions or the format used for responses. After discussion with the EORTC Translation Unit, we modified the Chinese wording in item 50 to ensure that the meaning of “lifeless” was clear. No changes were made to the remaining items.

**Conclusions:**

The simplified Chinese version of the EORTC QLQ-LC29 is now available on the EORTC website. This translation may contribute to the application of the EORTC QLQ-LC29 scale in both research and clinical practice in the Chinese population with lung cancer. Further evaluation of the psychometric properties of the translated EORTC QLQ-LC29 is warranted.

## Background

Lung cancer is the most common cancer in men and the second most common cancer in women worldwide and in China [1, 2]. About 800,000 people are diagnosed with lung cancer each year in China [2]. The overall prognosis of patients with lung cancer is poor, with a 5-year survival rate of less than 21% [3]. The available treatments include surgery, radiotherapy, chemotherapy, targeted therapy, and immunotherapy [3]. However, these treatments improve survival time but come at a cost of impaired health-related quality of life (HRQOL) [4]. In order to promote patient-centred care, it is important to focus on patient-reported outcomes, such as HRQOL, in addition to traditional clinical outcomes [5, 6].

For patients with lung cancer, HRQOL is usually measured by the European Organisation for Research and Treatment of Cancer (EORTC) Quality of Life Core Questionnaire (QLQ-C30) in conjunction with the specific module for lung cancer (QLQ-LC13) [7–9]. However, the EORTC QLQ-LC13 was developed 27 years ago and does not include items that adequately assess the adverse effects of newly developed treatments [10–12]. For example, the EORTC QLQ-LC13 does not assess skin rash, which is the most common side effect of targeted therapy. Therefore, the EORTC has recently developed the EORTC QLQ-LC29 questionnaire, an updated version of the EORTC QLQ-LC13, to measure HRQOL in patients with lung cancer [13, 14].

The EORTC QLQ-LC29 contains 29 items, of which 12 were taken directly from the EORTC QLQ-LC13, and 17 were newly added. It consists of five multi-item scales (coughing, shortness of breath, hair problems, fear of progression, and surgery-related symptoms) and 15 items related to single symptoms or side effects (coughing up blood, sore mouth or tongue, problems swallowing, tingling hands or feet, chest pain, pain in arm or shoulder, pain in other parts of the body, allergic reactions, burning or sore eyes, dizziness, splitting fingernails or toenails, skin problems, problems speaking, decrease in physical capabilities, weight loss problem), with a recall time of one week [13, 14]. All 29 items are rated using a 4-point Likert scale (1, ‘not at all’; 2, ‘a little’; 3, ‘quite a bit’; 4, ‘very much’). However, this updated questionnaire is not yet available in simplified Chinese. Given the large number of patients with lung cancer in China, the use of an updated questionnaire to measure HRQOL may benefit many patients as well as pave the way for future studies on HRQOL in these patients. Therefore, we translated the EORTC QLQ-LC29 questionnaire from English into simplified Chinese and pilot-tested its suitability for use in Chinese patients with lung cancer.

## Methods

**Fig. 1:**
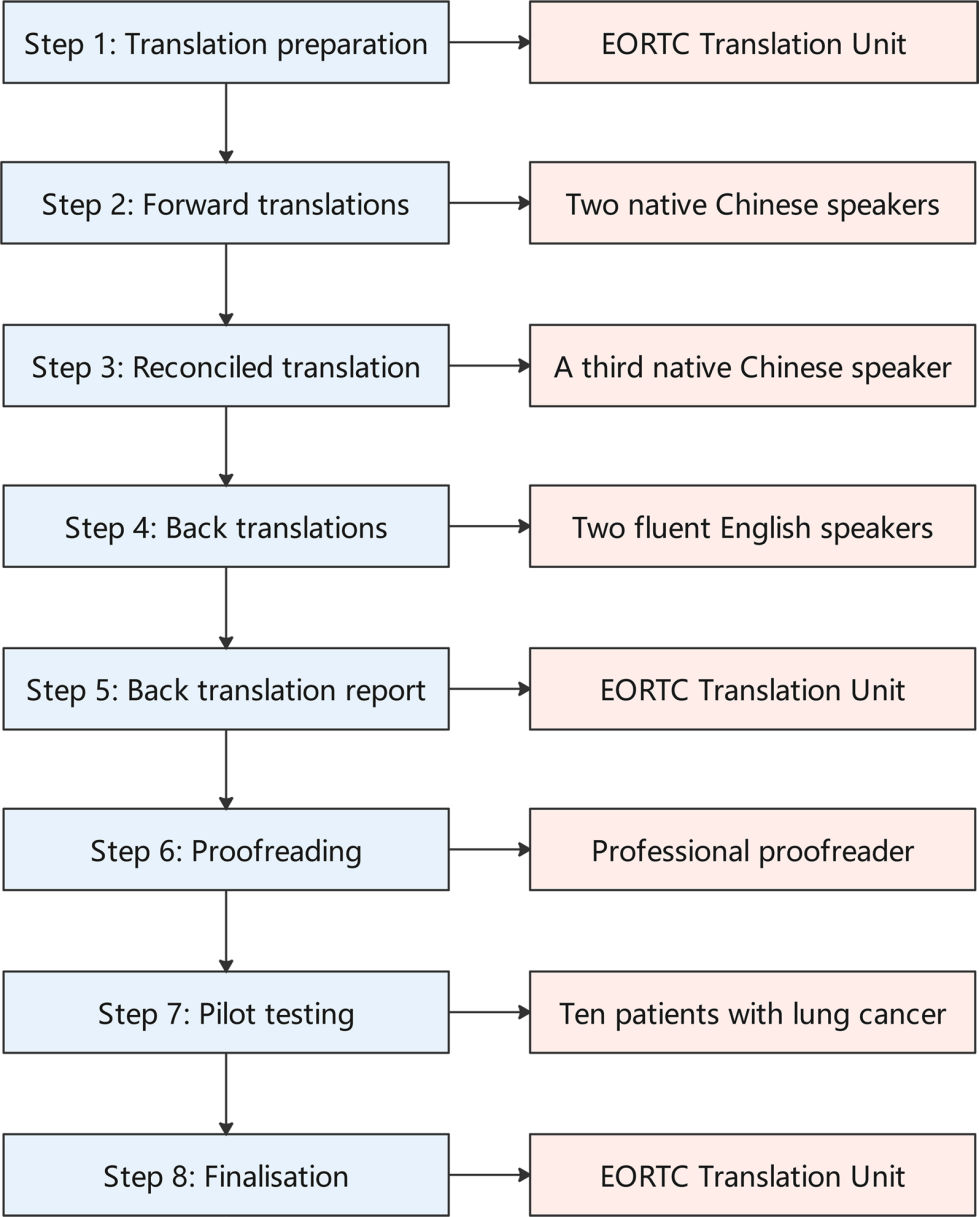
Process used to translate and adapt the EORTC QLQ-LC29 into simplified Chinese. EORTC, European Organisation for Research and Treatment of Cancer; QLQ-LC29, Quality of Life Questionnaire-Lung Cancer 29

We followed the EORTC translation manual [15], and the translation and adaptation process consisted of eight steps (Fig. 1).

### Step 1: translation preparation

Before officially starting the translation work, we sought permission from the EORTC Translation Unit to translate the questionnaire. When the application was approved, we obtained the translation files, including an English version of the EORTC QLQ-LC29 questionnaire, a translation manual, and a translation review report containing an original English version and some previous EORTC translations from the EORTC Item Library (a database of EORTC questionnaire items and their translations).

### Step 2: forward translations

Two native Chinese speakers (a thoracic surgeon and a thoracic nurse) with a good command of English independently translated the English version of the EORTC QLQ-LC29 into Chinese. Before translation, they received the English version of the EORTC QLQ-LC29 questionnaire and the file containing some existing translations from the EORTC Item Library.

### Step 3: reconciled translation

A third native Chinese speaker (the translation coordinator) reconciled the two forward translations into a best single translation for each item and made comments on the reconciliation process. The methods and criteria used were adopted from the translation manual [15].

### Step 4: back translations

Two PhD candidates (a native Chinese speaker studying in Sweden and an Egyptian studying in China) who are fluent in English independently translated the reconciled translated version into English. The two translators only received the reconciled translation and the instructions for back translation.

### Step 5: back translation report

The translation coordinator sent the five above-mentioned translation files (two forward translations, one reconciled translation and two back translations) together with the comments from the translation coordinator to the EORTC Translation Unit for a comprehensive review. After several rounds of discussion with the EORTC Translation Unit, a consensus was reached. A preliminary translation was then prepared by the EORTC Translation Unit for proofreading.

### Step 6: proofreading

The EORTC Translation Unit sent the preliminarily translated version of the EORTC QLQ-LC29 to a professional proofreader for review. The preliminary translation was compared with the original English questionnaire by the proofreader, who then prepared a report explaining all the changes and suggestions together, providing explanations of why they were needed. After all the questions had been discussed, and an agreement was reached between the translation coordinator and the proofreader, the Translation Unit prepared an interim translation for pilot testing.

### Step 7: pilot testing

The pilot testing was conducted in a tertiary cancer hospital located in southwest China. Participants in the pilot test were required to be native speakers of the Chinese language, to have a histological diagnosis of lung cancer, to be undergoing active cancer therapy, and to be able to provide informed consent. According to the EORTC translation manual, 10–15 subjects are required for pilot testing [15]. We selected 10 patients of varying age, sex, education level, and annual income to maximise the representativeness of the patient sample. Patients first completed the EORTC QLQ-LC29 questionnaire, then a semi-structured interview using a predefined EORTC template (a patient response sheet) was conducted by a well-trained interviewing researcher. Each patient was asked if any item was difficult to answer, confusing, difficult to understand, or upsetting. If so, the patient was encouraged to rephrase the question using their own words. The interviewer also tested some alternative wordings to check if they could make the items more easily understood. A report containing the results of the pilot testing and relevant comments was then sent to the EORTC Translation Unit for review.

### Step 8: finalisation

After several rounds of discussion, a consensus was reached between the translation coordinator and the EORTC Translation Unit. The Translation Unit approved the final translated version and closed the project.

### Statistical analysis

Simple descriptive statistics were used, including the frequency and median (range) for age, sex, educational level, type of medical insurance, personal annual income, employment status, type of current treatment, and time interval between the date of the interview and the most recent treatment. Quantitative information was presented in tabular form.

## Results

### Forward, reconciled, and back translations

We obtained the permission to perform the EORTC QLQ-LC29 translation work on November 17, 2020. Then, it took us 3 weeks to conduct the first round of forward translations, reconciled translation, and back translations. After that, we sent the “back translation report” to the EORTC Translation Unit. However, they reminded us that there are some existing translations and we should use them whenever possible. Thus, we reconciled the translations again and discussed them with the EORTC Translation Unit for subsequent two rounds. Finally, the existing translations from the EORTC Item Library were used for 19 items, and 10 items without existing translations were translated from scratch (items 43, 46, 49, 52, 53, 55, 56, 57, 58, and 59).

### Proofreading

The professional proofreader suggested minor modifications for three items (items 32, 47, and 50), which were existing translations. We accepted two proposed changes. One was for item 47 (skin problems), which had a formatting problem. The other one was item 50 (thin or lifeless hair) in which a word was mistranslated. For item 32 (coughing up blood), we suggested an alternative translation to make it more understandable for a layperson, which was finally accepted by the proofreader.

### Pilot testing

#### Patient characteristics

Ten patients were included in the pilot study. The median time interval between the interview date and the most recent treatment was 4 days (range 0–406 days). The median age was 64 years (range 31–69 years). Table 1 shows the patient characteristics. Fifty percent of the patients were aged 65 years or older, were male, had an educational level of high school or above, had a personal annual income ˂ 50,000 RMB, or were retired. Six patients had undergone surgery, three had received chemotherapy, and one had received radiotherapy.

**Table 1:** Patient characteristics (n = 10)

Variable	Number
Age, years	
< 65	5
≥ 65	5
Sex	
Male	5
Female	5
Educational level	
Less than high school	5
High school or above	5
Personal annual income, RMB	
< 50,000	5
50,000–100,000	3
> 100,000	2
Employment status	
Unemployed	2
Employed	3
Retired	5
Current treatment	
Surgery	6
Chemotherapy	3
Radiotherapy	1

#### Comments from patients, the translation coordinator, and the EORTC

As shown in Table 2, eight items (27.6%) received comments from patients (six items by one patient alone and the other two items, 35 and 50, by three patients). No patient commented on the instructions or the format used for responses. One typical comment from patients was that they did not know how to answer certain items concerning activities or symptoms that they had not recently experienced, such as climbing stairs (item 35), performing physical activities (item 53), and losing weight (item 54). Three patients undergoing chemotherapy expressed confusion about the chosen Chinese word for "lifeless" in item 50; because this word also contains a meaning of "angry" in Chinese. After discussing with the EORTC Translation Unit, we decided to modify the Chinese wording in item 50 to ensure that the meaning of “lifeless” was clear (Table 2). No changes were made to the remaining items. For the additional open-ended questions (items 60–62), one surgical patient reported sleeping disorders, and another patient who was receiving chemotherapy complained of nosebleeds and diarrhoea.

**Table 2:** Comments from patients, the translation coordinator, and EORTC on pilot testing

Item No	Item content	Comments from patients	Changes proposed by the translation coordinator	EORTC comments	Final results
35	Have you been short of breath when you climbed stairs?	One postoperative patient said that healthy people may also feel short of breath when climbing stairs, so the question should be whether the patient feels more shortness of breath compared to the time before treatment or before the illness Two postoperative patients said they had not climbed stairs in the last week, so they could not answer this question	No changes were required because only one postoperative patient mentioned this issue and two patients did not answer this question	Agreed, no change necessary	No change
39	Have you had hair loss?	One postoperative patient mentioned that healthy people also lose a small amount of hair every day, so the question should be whether the patient is losing more hair than usual due to treatment	We selected another translation from the existing translation column to make it more specific	The translation you selected is for the colorectal patients and the source is slightly different: Have you lost hair as a result of your treatment? Seeing as 'as a result of your treatment' is not in the source of this questionnaire, I suggest we keep the translation as it is. It should be clear from the context what it is asking, and the comment only comes from one patient	No change
49	Have you been afraid of tumour progression?	One patient undergoing radiotherapy said she did not know her disease was a tumour, so she was a bit confused when she read this question	No changes were required because most patients are aware of their cancer diagnosis nowadays	Agreed, no change necessary	No change
50	Have you had thin or lifeless hair as a result of your disease or treatment?	Three patients undergoing chemotherapy were confused by the chosen Chinese word for “lifeless” ("无生气"); because this word also contains a meaning of “angry” in Chinese	Use another Chinese word (“无生机”) to represent “lifeless” to avoid the ambiguity. Fifty percent of the patients thought the reworded version was better, and the other 50% considered both words were acceptable	For the sake of clarity, you mention that 50% of patients agreed with the change. Did the other 50% disagree and prefer the previous version? Your opinion on whether the change is better would be useful	Replace “无生气" with "无生机"
53	Have you experienced a decrease in your physical capabilities?	One patient undergoing chemotherapy said he did not know how to answer this question because he has not undertaken any physical activity recently	No changes were required because only one patient mentioned this issue	Agreed, no change necessary	No change
54	Has weight loss been a problem for you?	One patient undergoing chemotherapy said he had no weight loss and did not know how to answer this question	Almost all questions in the EORTC QLQ-LC29 are about what symptoms or problems patients have rather than how patients feel about symptoms that they may not have at all. Therefore, we recommend asking patients directly if they have any symptoms or problems, which is consistent with the format of the previous questions	Thank you for your input. I am afraid that we cannot change the wording of the original questionnaire, and the translation must match the source. If patients have not had weight loss, they can select 'not at all'. No change to the translation	No change
56	Has the area of your wound been oversensitive?	One postoperative patient mentioned that the word "oversensitive" was a little difficult to understand	No changes were required because only one postoperative patient mentioned this issue	OK, no change	No change
57	Have you been restricted in your performance due to the extent of surgery?	One postoperative patient mentioned that the meaning of the word “performance” was vague. Does it refer to living ability or daily activities?	“Daily activities” would be more specific than “performance”	If the majority of patients understood, is a change necessary? “Performance” relates to abilities/strength and how well particular tasks can be performed. This is a bit different to 'daily activities'. Seeing as only one patient mentioned this, I suggest no change	No change

#### Quantitative results

Three responses were missing. Two occurred in item 35 because two patients filled in the questionnaires on the third day after surgery and reported that they had not climbed stairs during the previous week. Another one occurred in item 57 because one postoperative patient felt the word “performance” in item 57 was difficult to understand. As shown in Table 3, the most frequently reported symptom was coughing, which was reported by eight patients. No patient reported allergic reactions, burning or sore eyes, or splitting of the fingernails or toenails. Three patients reported severe coughing, pain in the arm or shoulder, and worry about health in the future (each checked as “very much”). For the five items related to surgery, the most frequent complaint was pain in the area of surgery, which was reported by five patients.

**Table 3:** Quantitative results of pilot testing

Item No	Item content	Response options, number				Missing, number
		Not at all	A little	Quite a bit	Very much	
31	Have you coughed?	2	7	0	1	0
32	Have you coughed up blood?	7	2	1	0	0
33	Have you been short of breath when you rested?	9	1	0	0	0
34	Have you been short of breath when you walked?	7	3	0	0	0
35	Have you been short of breath when you climbed stairs?	4	4	0	0	2
36	Have you had a sore mouth or tongue?	8	2	0	0	0
37	Have you had problems swallowing?	9	1	0	0	0
38	Have you had tingling hands or feet?	8	2	0	0	0
39	Have you had hair loss?	6	2	2	0	0
40	Have you had pain in your chest?	5	4	1	0	0
41	Have you had pain in your arm or shoulder?	5	4	0	1	0
42	Have you has pain in other parts of your body?	7	3	0	0	0
43	Have you had allergic reactions?	10	0	0	0	0
44	Have you had burning or sore eyes?	10	0	0	0	0
45	Have you been dizzy?	6	4	0	0	0
46	Have you had splitting fingernails or toenails?	10	0	0	0	0
47	Have you had skin problems (e.g., itchy, dry)?	6	4	0	0	0
48	Have you had problems speaking?	9	1	0	0	0
49	Have you been afraid of tumour progression?	3	5	2	0	0
50	Have you had thin or lifeless hair as a result of your disease or treatment?	8	2	0	0	0
51	Have you worried about your health in the future?	4	4	1	1	0
52	Have you had dry cough?	4	5	1	0	0
53	Have you experienced a decrease in your physical capabilities?	4	5	1	0	0
54	Has weight loss been a problem for you?	7	2	1	0	0
55	Have you had pain in the area of surgery?	1	4	1	0	0
56	Has the area of your wound been oversensitive?	3	3	0	0	0
57	Have you been restricted in your performance due to the extent of surgery?	3	2	0	0	1
58	Have you had any difficulty using your arm or shoulder on the side of chest operation?	2	3	1	0	0
59	Has your scar pain interfered with your daily actives?	2	4	0	0	0

#### Finalisation

After two rounds of discussion with the EORTC Translation Unit, the translation project was finalised on February 1, 2021. The final simplified Chinese version of the EORTC QLQ-LC29 is available on the EORTC website (https://qol.eortc.org/questionnaires).

## Discussion

In this study, we translated the EORTC QLQ-LC29 from English into simplified Chinese and pilot-tested the translated questionnaire. The EORTC QLQ-LC29 is designed to assess HRQOL in patients with lung cancer and has updated the EORTC QLQ-LC13 by including more items related to targeted therapy and surgery [13, 14]. Our translation work provided a foundation for potential future application of this questionnaire in Chinese patients with lung cancer.

As in similar projects [16, 17], we adhered strictly to the EORTC translation guidelines and all the steps were audited by the EORTC Translation Unit. During the translation process, we encountered some difficulties and gained some experience. We share our personal experience here in the hope that it may help other research teams intending to translate EORTC scales or similar questionnaires. First, following the EORTC translation guidelines and maintaining constant communication with the Translation Unit are essential to the smooth implementation of the translation process, given that less experienced translators may overlook some details. For example, during the forward and reconciled translation phase, we spent a considerable amount of time searching for appropriate wording. Later, the Translation Unit reminded us that we should use the existing translations as much as possible, provided that they are understandable and grammatically consistent. This recommendation not only facilitated the forward translation but also ensured the comparability and consistency between the different EORTC questionnaires. Second, if the literal translation is understandable for most of the patients, it is preferable to retain the literal translation rather than use a paraphrasing translation. For example, during the pilot testing, only one patient was confused about the meaning of the translated word for “performance” in item 57 (Have you been restricted in your performance due to the extent of surgery?). It is difficult to find a Chinese word that has a meaning identical to the English word “performance”, and the chosen Chinese word was based on a literal translation. Although it may not convey the original meaning perfectly, we decided to adopt it because there was no better alternative wording. Third, to avoid ambiguity, it is important to keep in mind that some of the Chinese words may have more than one meaning. For example, there are two Chinese words that convey the meaning of “lifeless”. The word that we originally chose (“无生气”) also contains a meaning of “angry”, which was confusing for patients. Therefore, we replaced it with another similar but unequivocal word (“无生机”). Fourth, it would be preferred that the back translation is performed by native English speakers, although this is not mandatory [15]. In this study, the two back translators are not native speakers of English, although they have a good command of English. Compared with native English translators, this may lead to some deviations due to different cultural backgrounds. This may be why the EORTC requires that the Translation Unit reviews the back translations and discusses all questions with the translation coordinator repeatedly to reach a consensus.

Assessment of the comprehensibility of the EORTC QLQ-LC29 after translating into another language is one of the most important parts of the translation project. Pilot testing aims to assess the items, instructions, and response format of a scale for clarity [18]. During our pilot testing, eight items (27.6%) received comments from patients. The percentage was relatively high compared with that in the pilot testing studies of the EORTC QLQ-LC29 in other languages [16, 17]. This may be due to cultural differences. For the six items that were commented on by only one patient, we decided to keep their translations unchanged. For item 35, two postoperative patients reported that they had not climbed stairs during the previous week, so they did not know how to answer this question. This may be because these two patients only started minor mobility as they were in the immediate postoperative stage. However, given that the increasing popularity of lifts and escalators decreases the likelihood of climbing stairs, this input from patients may suggest a potential update for this item in the future.

The results of the pilot testing of the simplified Chinese version of the EORTC QLQ-LC29 indicated that the symptom burden was low in Chinese patients with lung cancer [17]. A possible reason for this finding is that most of the enrolled patients had either just started lung cancer treatment or had completed the treatment for some time. Furthermore, the representativeness of our results is limited because of the very small sample size (10 patients).

## Conclusions

This study reports the process involved in translating the EORTC QLQ-LC29 into simplified Chinese and pilot testing it in Chinese patients with lung cancer. The simplified Chinese version of the EORTC QLQ-LC29 is now available on the EORTC website (https://qol.eortc.org/questionnaires). This translation paves the way for future psychometric studies on the translated questionnaire, and may contribute to the application of the EORTC QLQ-LC29 in research and clinical practice in the Chinese population with lung cancer.

## Data Availability

The full study data are available from the corresponding author upon reasonable request.

## Abbreviations

HRQOL: Health-related quality of life
EORTC: European Organisation for Research and Treatment of Cancer
QLQ-LC13: Quality of Life Questionnaire-Lung Cancer 13
QLQ-LC29: Quality of Life Questionnaire-Lung Cancer 29
